# Efficacy of a modern neuroscience approach versus usual care evidence-based physiotherapy on pain, disability and brain characteristics in chronic spinal pain patients: protocol of a randomized clinical trial

**DOI:** 10.1186/1471-2474-15-149

**Published:** 2014-05-08

**Authors:** Mieke Dolphens, Jo Nijs, Barbara Cagnie, Mira Meeus, Nathalie Roussel, Jeroen Kregel, Anneleen Malfliet, Guy Vanderstraeten, Lieven Danneels

**Affiliations:** 1 Department of Rehabilitation Sciences and Physiotherapy, Ghent University, Campus Heymans (UZ, 3B3), De Pintelaan 185, 9000 Ghent, Belgium; 2 Pain in Motion Research Group, Departments of Human Physiology and Physiotherapy, Faculty of Physical Education & Physiotherapy, Vrije Universiteit Brussels, Brussels, Belgium; 3 Department of Physical Medicine and Physiotherapy, University Hospital Brussels, Brussels, Belgium; 4 Pain in Motion Research Group, Rehabilitation Sciences and Physiotherapy, Faculty of Medicine and Health Sciences, Universiteit Antwerpen, Antwerpen, Belgium; 5 Department of Physical and Rehabilitation Medicine, Ghent University, Ghent, Belgium

**Keywords:** Chronic pain, Low back pain, Neck pain, Education, Exercise, Motor control, Neuroscience, Randomized controlled trial

## Abstract

**Background:**

Among the multiple conservative modalities, physiotherapy is a commonly utilized treatment modality in managing chronic non-specific spinal pain. Despite the scientific progresses with regard to pain and motor control neuroscience, treatment of chronic spinal pain (CSP) often tends to stick to a peripheral biomechanical model, without targeting brain mechanisms. With a view to enhance clinical efficacy of existing physiotherapeutic treatments for CSP, the development of clinical strategies targeted at ‘training the brain’ is to be pursued. Promising proof-of-principle results have been reported for the effectiveness of a modern neuroscience approach to CSP when compared to usual care, but confirmation is required in a larger, multi-center trial with appropriate evidence-based control intervention and long-term follow-up.

The aim of this study is to assess the effectiveness of a modern neuroscience approach, compared to usual care evidence-based physiotherapy, for reducing pain and improving functioning in patients with CSP. A secondary objective entails examining the effectiveness of the modern neuroscience approach versus usual care physiotherapy for normalizing brain gray matter in patients with CSP.

**Methods/Design:**

The study is a multi-center, triple-blind, two-arm (1:1) randomized clinical trial with 1-year follow-up. 120 CSP patients will be randomly allocated to either the experimental (receiving pain neuroscience education followed by cognition-targeted motor control training) or the control group (receiving usual care physiotherapy), each comprising of 3 months treatment. The main outcome measures are pain (including symptoms and indices of central sensitization) and self-reported disability. Secondary outcome measures include brain gray matter structure, motor control, muscle properties, and psychosocial correlates. Clinical assessment and brain imaging will be performed at baseline, post-treatment and at 1-year follow-up. Web-based questionnaires will be completed at baseline, after the first 3 treatment sessions, post-treatment, and at 6 and 12-months follow-up.

**Discussion:**

Findings may provide empirical evidence on: (1) the effectiveness of a modern neuroscience approach to CSP for reducing pain and improving functioning, (2) the effectiveness of a modern neuroscience approach for normalizing brain gray matter in CSP patients, and (3) factors associated with therapy success. Hence, this trial might contribute towards refining guidelines for good clinical practice and might be used as a basis for health authorities’ recommendations.

**Trial registration:**

ClinicalTrials.gov Identifier: NCT02098005.

## Background

Chronic spinal pain (CSP) is a major public health problem worldwide as it is a common disorder and a major cause of disability and health care utilization [1-4]. Taking chronic non-specific low back pain as an example, best estimates suggest that its prevalence is about 23% with 11-12% of the population being disabled by it [2,5]. According to the Global Burden of Disease Study 2010 [4], low back pain contributes 83.1 million years lived with disability (i.e., 10.7% of total years lived with disability), thereby being the leading cause of years lived with disability. From an economic perspective, the group of chronic, disabling patients is responsible for the bulk of low back pain care resource consumption, denoting considerable costs [6,7]. No wonder, then, that research on the most effective and affordable strategies to deal with CSP has been strongly advocated [1-3,8].

Management of CSP should aim at achieving and maintaining a clinically important reduction in pain and disability with a minimum amount of costs and inconveniences related to the intervention [2,9]. For CSP, most clinical practice guidelines agree on the use of brief education about the problem, recommendations to stay active, adjunctive analgesics, non-steroidal anti-inflammatory drugs, weak opioids (short-term use), exercise therapy (of any sort), spinal manipulation, multidisciplinary rehabilitation, cognitive behavioral therapy, and strong opioids [2,5,10,11]. Systematic reviews of the most commonly applied treatments for CSP in primary care generally reach similar conclusions: most treatments provide small, short-term benefits when compared to no or sham treatment, but offer little benefit when compared to other forms of intervention [8,12-18]. In an attempt to account for the equivalence in outcome of very disparate treatments, Wand and O’Connell [19] suggested that distinct treatments might show similar effectiveness because they could all work through the same mechanism, e.g. by affecting higher neurological levels. As such, one could hypothesize that greater effect sizes may be observed if treatment strategies would focus more on central processes [2,19].

In non-specific CSP, there is increasing evidence for supraspinal abnormalities (i.e., distinct brain activity and morphology, hyperexcitability of the central nervous system and central sensitization) in addition to the compelling evidence for impaired motor control of spinal muscles (reviewed elsewhere [19-24]). As such, the development of novel clinical strategies targeting at normalizing neurological processing (“training the brain”) to achieve pain reduction and improved function has been argued to be a challenging new direction for musculoskeletal clinicians and researchers involved in the management of CSP. Still, at present, physiotherapy for patients with CSP is often based either on a pure biomedical (e.g. neuromuscular training) or psychosocial model (e.g. graded exposure in vivo, graded activity, multidisciplinary pain treatment). Yet neither approaches account for our current understanding of modern pain neuroscience. Therefore, the theoretical rationale for combining both approaches in a program that addresses central nervous system dysfunctions (e.g. dysfunctional endogenous analgesia [25], central hyperexcitability [26]), psychosocial factors (e.g. pain catastrophizing [22] and illness perceptions [27]) as well as peripheral dysfunctions (impaired motor control of spinal muscles) in a broader biopsychosocially-driven framework has been elaborated in a recent perspective paper of our research group [22].

Addressing the proof-of-concept, 1 single case study [28] and 2 small-scale single-centered randomized controlled trials [29,30] support the clinical effectiveness (large effect size and small numbers needed to treat) of the modern neuroscience approach to CSP (i.e., pain neuroscience education followed by cognition-targeted motor control training) compared to usual care in terms of pain reduction and improved function, and suggest that side effects are not to be expected. However, these pilot studies comprised relatively small populations of low back pain patients who were treated with the modern neuroscience approach and follow-up after 1 year was reported for less than 20 patients. In addition, these studies were from the same research group and from 1 single treatment center. Hence, replication in a larger, multi-center trial with appropriate evidence-based control intervention performed by researchers who are independent from the research group who generated the proof-of-concept, is required. Preferably, such a project does not only focus on chronic low back pain, but on CSP in general, including non-traumatic chronic low back and neck pain, failed back surgery and chronic whiplash associated disorders.

Previous work from our research group has shown that therapeutic pain neuroscience education alone is able to improve brain-orchestrated endogenous analgesia in patients with chronic widespread pain [31]. Until now, no brain imaging studies have evaluated whether (and how) physiotherapy can influence brain characteristics in patients with CSP. Hence, this will be the first randomized trial examining whether a treatment targeted at the brain actually does alter the brain’s characteristics. In addition, the identification of major reasons for (sub) optimal treatment success or subgroups that benefit most can help the physician to optimize CSP management.

Based on this background, this 12-month prospective study has been designed in order to estimate the effectiveness of a modern neuroscience approach in CSP patients, its determinants and its changes during a 1-year follow-up.

### Objectives

Table 1 summarizes the primary and secondary objectives of the study. The primary objectives are to investigate whether treatment with a modern neuroscience approach in CSP patients results in a significant decrease in pain and disability compared to usual care evidence-based physiotherapy.

**Table 1:** Primary and secondary objectives to be investigated in CSP patients

**Primary objectives**	**Longitudinal phase**
	- Effect of a modern neuroscience approach on pain compared to usual care evidence-based physiotherapy
	- Effect of a modern neuroscience approach on indices of central pain processing (i.e. widespread cold pain, pressure pain tresholds (PPTs) and conditioned pain modulation) compared to usual care evidence-based physiotherapy
	- Effect of a modern neuroscience approach on functioning compared to usual care evidence-based physiotherapy
**Secondary objectives**	**Cross-sectional phase (baseline)**
	- Relation between brain gray matter structure (cortical thickness) and pain (including symptoms of central sensitization)
	- Relation between brain gray matter structure (cortical thickness) and (dysfunctional) motor control
	- Relation between pain and motor control
	- Associations between pain, functional disability, and physical/psychological correlates of pain and dysfunctioning
	**Longitudinal phase**
	- Effect of a modern neuroscience approach on brain gray matter structure compared to usual care evidence-based physiotherapy
	- Effect of a modern neuroscience approach on motor control compared to usual care evidence-based physiotherapy.
	- Relation between changes in pain, functional disability, and physical/psychological correlates of pain and dysfunctioning
	- Proportion of patients that reach therapy success after 3, 6 and 12 months from cross-sectional phase visit
	- Factors associated with clinically important changes in primary outcome measures
	- Factors associated with poor outcome following treatment
	- Mediating factors for treatment effects

CSP: chronic spinal pain.

## Methods/Design

### Design

**Figure 1:**
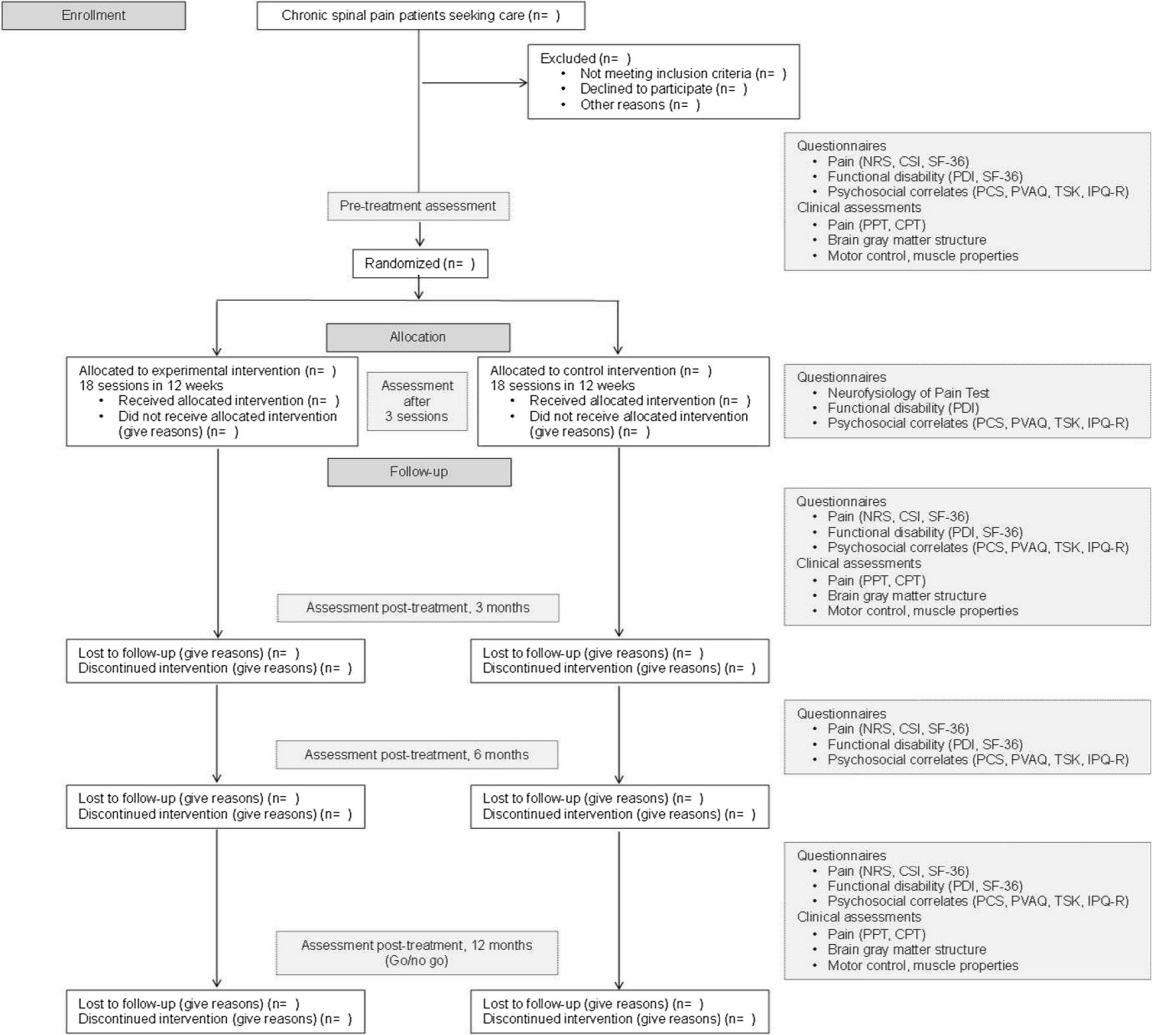
**Flow chart of research design.** CPT: cold pressor test; CSI: Central Sensitization Inventory; IPQ-R: Illness Perception Questionnaire-Revised; NRS: Numerical Rating Scale; PCS: Pain Catastrophizing Scale; PDI: Pain Disability Index; PPT: pressure pain threshold; PVAQ: Pain Vigilance and Awareness Questionnaire; SF-36: medical outcomes Short Form 36 Health Service Survey; TSK: Tampa Scale for Kinesiophobia.

The present study is a 12-month multi-center, triple-blind, randomized, controlled, parallel group trial that will be carried out between February 2014 and April 2017. Patients with CSP (including low back and neck pain, failed back surgery and chronic whiplash associated disorders) will be enrolled in a structured 3-month rehabilitation program organized in 2 university hospitals in Belgium (Ghent University Hospital and University Hospital Brussels). More specifically, therapeutic pain neuroscience education combined with cognition-targeted motor control training will be compared to back/neck school and general exercises. Treatment outcomes will be assessed at baseline, after 3 treatment sessions, post-treatment (at 3 months), at 6 months and 1 year follow-up (Figure 1). Following the go/no-go principle, however, the 1-year follow-up examination will not take place in case that treatment effects are no longer present at 6 months follow-up in none of the treatment arms.

### Study population

The study population will include approximately 120 CSP patients. Patients will be recruited by the participating research groups (Ghent and Brussels) from the hospital, from primary care practices (medical doctors) and via adverts. Dutch speaking male and female adult (aged 18 – 65 years) patients with non-specific CSP (at least 3 days/week) for at least 3 months, currently seeking care for low back or neck pain, not starting new treatments or medication and continuing usual care 6 weeks prior to and during study participation (to obtain a steady state), will be eligible for study participation after signing the informed consent. Patients with neuropathic [32] or chronic widespread pain as defined by the criteria of the 1990 ACR [33] will be excluded. A history of back or neck surgery in the past 3 years, a lifetime history of specific back or neck surgery (e.g. surgery for spinal stenosis) or osteoporotic vertebral fractures, rheumatologic diseases, concomitant therapies (i.e., rehabilitation, alternative medicine or therapies) and medical conditions or contra-indications for MRI are also exclusion criteria. Pregnant women and women given birth in the last year before enrolment will be excluded from the study, as are patients who live and work outside a 50-km radius of the treatment location. Study participants will be asked to refrain from analgesics 48 h prior to assessments, to abstain from caffeine, alcohol and nicotine 24 h prior to assessment, and not to undertake physical exercise (>3 metabolic equivalents) in the 3 days before assessment.

### Baseline assessment and randomization

After eligibility has been confirmed, patients will be informed about the study comparing two physiotherapeutic treatment options. After obtaining written informed consent, baseline measurements will be performed (see next paragraph). Participants will then be randomized to either control or experimental group (1:1 ratio) using a stratified permuted block allocation with stratification factors being treatment center (Ghent or Brussels), dominant pain location (low back or neck) and gender (male or female) and with a block size of four [34,35]. Randomization will be done at the Biostatistics Unit (Ghent University) by an independent investigator using the SAS version 9.4 package. The randomization schedule will be known only to 1 investigator who is not involved in recruiting participants. The randomization will be concealed from patients and the other investigators involved in patient assessments and analyses.

### Outcome measures

Pain and restrictions in functioning will be the primary outcome measures. Secondary outcome measures will include brain gray matter structure, motor control, muscle properties, and psychosocial factors that may interfere with pain. Methods for assessment will include web-based questionnaires (Dutch version), clinical testing and brain imaging using magnetic resonance imaging (MRI).

### Primary outcomes

Pain, including symptoms and indices of central sensitization, will be assessed through a self-reported web-based questionnaire and a clinical assessment. The following *self-administered online tools* will be used for pain assessment at baseline, post-treatment, and at 6 and 12-months follow-up:

A Numerical Rating Scale (NRS) for pain ranging from 0 = “no pain” to 10 = “the worst pain imaginable” (“How would you rate your spinal pain, on average, over the last three days?”) and an additional enquiry about the number of health visits for spinal pain over the course of the follow-up period: “Since your assessment on [date of final assessment], how many times have you consulted a health care professional for your spinal pain?” [29],

the Central Sensitization Inventory (CSI) [36], comprising 2 parts: current health symptoms indicative of central sensitization (25 statements; responses are recorded about the frequency of each symptom, with a Likert scale from 0 (never) to 4 (always), resulting in a total possible score of 100; higher scores are associated with a higher degree of self-reported symptomology) and previously diagnosed central sensitivity syndromes and related conditions [36],

the SF-36 (Short Form Health Survey – 36 item), see below.

*Clinical assessment* of pain will comprise pressure pain threshold (PPT) measurements and the cold pressor test (CPT). Measurements will be taken at baseline, post-treatment, and at 1-year follow-up.

Pressure algometry will be used to measure PPTs at the symptomatic levels (the upper trapezius muscle midway between C7 and the tip of the acromion [37] and 5 cm lateral of the spinous process of L3 [38]) and at remote sites (quadriceps muscle and the web between thumb and index finger [39]) using a digital Wagner algometer (Wagner Instruments, Greenwich, CT). The rate of pressure increase will be maintained at a constant rate of 1 kg/m^2^/s [38,40]. PPTs will be tested unilaterally: the most painful side will be assessed unless the pain is evenly distributed on both sides. Then, the dominant side will be investigated. At each of the selected measuring points, the threshold will be determined as the mean of 2 consecutive (30 s in between) measurements [40]. By evaluating symptomatic and remote sites, both primary and secondary hyperalgesia can be assessed [41-43]. Algometry has been shown to provide a reliable and valid measure of PPTs [44].

The CPT, a widely used and reliable test, will be used to evaluate the efficacy of the descending inhibitory modulation of pain (i.e. conditioned pain modulation) [45]. The conditioning stimulus in our diffuse noxious inhibitory control will be realized by the immersion of the contralateral hand to the PPT measurements (up to the wrist; see above) for 2 minutes into a tub containing 12 degrees C cold water [46]. Before and during submersion, the PPTs will be measured on several body sites using pressure algometry (see above; noxious mechanical test stimuli). Subjects will be asked to rate the perceived pain intensity on an 11-point visual NRS after 30 and 115 seconds.

Spinal pain related restrictions in functioning will be assessed using self-reported web-based questionnaires:

The social disability associated with spinal pain will be assessed by the Pain Disability Index (PDI) [47,48], consisting of 7 items to be rated on an 11-point NRS (range from 0 = “no disability” to 10 = “total disability”). The degree to which pain interferes with the performance of social roles in 7 areas will be evaluated: family/household responsibilities, recreation, social activities, occupation, sexual behavior, self care, and life support activities. The sum score will be used in this project; higher scores indicate more disability. Patients will complete the PDI at baseline, after the first 3 treatment sessions, post-treatment, at 6-month follow-up and at 1-year follow-up. Results in different chronic pain populations indicate that the PDI is a reliable and valid instrument [49].

The Short Form 36 Health Status Survey (SF-36) will be used to assess functional status and well-being or quality of life at baseline, post-treatment, and at 6 and 12-months follow-up. The SF-36 contains 8 dimensions (physical functioning, social functioning, physical role, emotional role, mental health, vitality, bodily pain, and general health perceptions). The overall value ranges from 0 to 100, with improvement as scores increase. The psychometric properties of the SF-36 are well-characterized in a wide variety of patient populations [50,51].

### Secondary outcomes

#### Brain gray matter structure

High-resolution MR scanning will be performed on a 3 T Trio Tim magnet (Siemens medical solutions, Erlangen, Germany) with a standard head coil. Using voxel-based morphometry, brain gray matter structure will be investigated in brain areas involved in pain processing and motor control. MRI data will be obtained at baseline, post-treatment, and at 1-year follow-up.

More specifically, a T1 weighted structural MRI will be acquired by using a 3D-FLASH sequence (repetition time 2250 ms, echo time 4.18 ms, flip angle 9°, field of view 256 × 256; 176 slices), acquisition time 05′14″. Regional gray matter density will be assessed with voxel-based morphometry that allows for applying voxelwise statistics to detect regional differences in gray matter volumes. Preprocessing will involve spatial normalization, gray matter segmentation, and 10 mm spatial smoothing with a Gaussian kernel [52]. The T1-weighted images will be processed and analyzed with FreeSurfer; cortical thickness and surface area will be calculated.

#### Motor control

Motor control will be assessed by clinical measurements of postural steadiness, habitual standing posture, spinal range of motion, and sensorimotor control. These aspects will be tested at baseline, and at 3-month and 1-year follow-ups.

*Postural steadiness* will be characterized by postural sway features as measured by an AccuGait portable forceplate (50 cm × 50 cm) (Advanced Medical Technology, Inc. Watertown, MA) during bipedal standing with eyes closed on a firm surface. Centre of pressure (COP) data will be sampled at a frequency of 100 Hz during 3 trials of 90 s. The following COP stability parameters will be computed: mean sway velocity, 95% confidence ellipse area, standard deviation of sway velocity, of medio-lateral COP data and of anterior-posterior COP data. During these posturography measurements, subjects will be barefoot and will be instructed to stand as still as possible with arms by their sides. Test-retest reliability of posturography is well-documented in adult populations [53]. In addition, each subject will complete a clinical balance test: standing in tandem stance (heel-to-toe) with either the left or right foot in front, with eyes open and eyes closed. Patients’ performance during a 30-second tandem stance will be graded as pass/fail [54].

For the assessment of *habitual standing posture in the sagittal plane*, the orientation of gross body segments with respect to the vertical will be quantified using post-hoc analyses of digitized photographs of participants. Retro-reflective markers will first be placed on the C7 spinous process, greater trochanter, and lateral malleolus by one trained examiner. Lateral photographs will then be taken within a standardized photographic set-up after each patient is asked to stand normally and relaxed, looking straight ahead. Using ImageJ software (National Institutes of Health, Bethesda, MD), the craniovertebral angle will be calculated in patients with dominant neck pain. In patients with dominant pain located in the low back, three angular measures will be determined: trunk lean angle, body lean angle and pelvic displacement angle. For more detailed methods, see previous articles by Dolphens et al. [55-57]. Furthermore, in low back pain patients, lumbar lordosis will be measured using a skin-surface hand-held electromechanical device, the Spinal Mouse (Idiag; Voletswil, Switzerland). The intratester, intertester and day-to-day reliability of this wheeled accelerometer have been published in previous studies [55,58-60].

*Range of motion* of the cervical spine (flexion, extension, lateral flexion) will be measured in neck pain patients (seated position) using the Acumar™ digital inclinometer (Model ACU 360, Lafayette Instrument Company, Lafayette, IN) that is placed on the vertex of the head through T1. According to the manufacturer’s specifications, this device is capable of measuring a range up to 180 degrees with an accuracy of ± 1 degree. In low back pain patients, the lumbar range of motion in the sagittal plane (flexion, extension) will be measured using the Spinal Mouse device (see above) with patients in the standing position. Furthermore, lumbar lateral flexion will be measured using the Acumar™ digital inclinometer (see above) placed on T12 through S1. For each movement direction, the mean of three consecutive measurements will be taken.

With regard to *sensorimotor control*, the following components will be assessed:

Proprioception will be determined by evaluating the position-reposition accuracy of the spine. In neck pain patients, repositioning will be assessed by the cervicocephalic relocation test to the neutral head position with eyes closed [61]. More specifically, patients will be seated on a stool without backrest with their hands on their thighs, and hips and knees bent 90 degrees. After an active submaximal range cervical flexion-extension and right and left rotation, patients will be instructed to relocate back to the neutral position. Absolute and relative errors will be expressed in degrees [62]. In low back pain patients, position-reposition accuracy will be assessed both in the sitting and standing position [63]. First, the tester will place the subject in a neutral lumbar spine position [63]. Then, after having performed three pelvic rotations (anterior and posterior pelvic rotation), the subject will be asked to reassume the reference position as accurately as possible. Assessment will be based on a clinical rating scale (unpublished results) evaluating the position-reposition accuracy to the neutral lumbar spine and pelvis position, and deviations in adjacent regions (thoracic kyphosis, trunk inclination, antero-posterior translation of the pelvis with respect to the feet (in standing position only), degree of knee flexion (in standing position only)) compared to the initial, neutral position. Each position-reposition cycle will be performed once.

Neuromuscular control will be assessed as the patients’ ability to perform the skill of activation of specific, deep stabilizing muscles for which there is scientific evidence that they play a crucial role in spinal stability. In neck pain patients, the contraction of the deep neck flexors will be evaluated through the craniocervical flexion test [64], and the lower and middle trapezius muscles will be assessed via the scapular holding test/scapula setting [65]. More specifically, assessment of contraction of the deep neck flexors will be scored based on the output obtained via an air-filled pressure sensor (Stabilizer, mmHg), substitution of superficial muscles, movement pattern and the holding capacity. Analogously, performance of the neuromuscular control of the scapulothopracic muscles will be based on the quality of contraction, substitution, movement pattern, and ability to maintain contraction as scored on a clinical rating scale (unpublished results). In low back pain patients, multifidus and transverse abdominis contraction will be evaluated in prone and supine (drawing-in action), respectively. Performance will be scored using a clinical rating scale based on the quality of contraction, substitution of superficial muscles, symmetry of contraction and the holding capacity.

Movement control of the lumbar spine will be assessed in low back pain patients. A set of 6 dissociation tests that have shown substantial reliability [k > 0.6] [66] will be included: 1) waiters bow (flexion of the hips in upright standing position without movement (flexion) of the low back); 2) pelvic tilt (active dorsal tilt of the pelvis in upright standing); 3) one leg stance (from normal standing to one leg stance: measurement of lateral movement of the belly button); 4) sitting knee extension (upright sitting with neutral lumbar lordosis; extension of the knee without movement (flexion) of low back); 5) rocking forward/backward (quadruped position). Starting position 90° hip flexion. Transfer of the pelvis backwards and forwards (“rocking”) keeping low back in neutral; 6) prone knee flexion (prone lying, active knee flexion). The order of the tests will be standardized. A strict protocol to instruct the tests and to rate test performances as “correct” or “incorrect” will be applied as described by Luomajoki et al. [66], resulting in an overall score between 0 and 6. No movement control test will be performed in neck pain patients.

Low back pain patients will perform a lumbopelvic control test in the sitting and standing position. A clinical rating scale (article submitted for publication) comprising the quality of the lumbopelvic motion, control of the adjacent areas, preference of motion direction, breathing, and repetitions will be used for evaluation, with higher scores indicating better performance.

As the observers’ level of experience is important for good test reliability, all tests will be rated by the same experienced observer [66-69].

### Muscle properties

The assessment of intrinsic muscle properties (i.e., muscle strength and endurance) will take place at baseline, post-treatment and at 1-year follow-up.

Isometric *muscle strength* will be measured with a hand-held dynamometer (MicroFet2; Hoggan Health Industries Inc., West Jordan, UT) with a sensitivity of 0.4 N. In neck pain patients, the testing procedure will consist of seated isometric strength measures for neck flexion, extension and side bending (left and right). For each movement, the dynamometer will be placed on specific marker points: the imaginary line through the supra-orbital notch for flexion, the protuberance of the occiput for extension and the temporal bone for side bending. In patients with dominant low back pain, trunk flexor and extensor muscle strength will be evaluated. Trunk flexor muscle strength will be measured with the patient in a semi-upright sitting position (45 degrees), knees extended, arms flexed alongside the trunk, hands placed on the homolateral shoulder, and head mid-line. The end piece of the dynamometer will be applied on the sternum at the centre of the chest. Trunk extensor muscle strength will be measured with the subject in prone, hands underneath the forehead and head mid-line. The dynamometer will be applied at the inferior angle of the scapulae at the centre of the back. For muscle strength tests, patients will be allowed 1 warm-up trial, followed by 3 successive maximal effort trials separated by 10-s rest periods. Patients will be asked to take 1 or 2 s to come to maximum effort and then, 5 s as forcefully as possible. The mean of 3 consecutive measurements per movement will be taken.

*Muscle endurance* will be assessed using isometric tests, i.e. patients will be instructed to maintain an imposed posture as long as possible. In neck pain patients, endurance of the neck flexors will be evaluated with the deep neck flexor endurance test [70,71]: patients in a supine, hook-lying position will be instructed to maximally tuck their chin, lift their head by approximately 2.5 cm and to hold this position as long as possible. Low back pain patients will perform a trunk flexor and extensor endurance test. The flexor endurance test will require subjects to hold their upper body in an unsupported, semi-upright sitting position (45 degrees) with knees extended, arms flexed alongside the trunk, and hands placed on the shoulders [63]. Isometric endurance of the back muscles will be assessed using the modified Biering-S∅rensen test [63,72,73]. For endurance tests, the position-holding time will be recorded. Verbal encouragement will be given by the tester during the endurance tests to ensure that the maximal effort is produced by the patient.

### Psychosocial correlates

Patients will complete a web-based online battery of questionnaires at baseline, after 3 treatment sessions, post-treatment, and at 6- and 12-month follow-ups. The following standardized and reliable questionnaires (Dutch version) will be used to measure psychological factors that may interfere with pain:

The Pain Catastrophizing Scale (PCS) will be included to assess catastrophic thinking about pain. It consists of 13 items describing different thoughts and feelings that individuals may have when experiencing pain. Items are scored on a 5-point scale. A general score and scores on 3 subscales (i.e., helplessness, magnification, and rumination) will be obtained; higher scores indicate more severe catastrophic thoughts about pain [74,75]. The psychometric properties of the Dutch version of the PCS are well established [74,76,77].

The Pain Vigilance and Awareness Questionnaire (PVAQ) will be used to investigate patients’ attention to pain. It is a 16-item measure of attention to pain that assesses awareness, consciousness, vigilance, and observation of pain. Scores range from 0 to 80 and high scores correspond to hypervigilance for pain. The items have demonstrated good internal consistency (Cronbach’s alpha = .86) in a population of chronic low back pain patients [78].

The Tampa Scale for Kinesiophobia (TSK) is a 17-item questionnaire that will be used to measure the fear of (re) injury due to movement [79,80]. Scores range from 17 to 68, with scores ≤ 37 suggesting low fear of movement and scores > 37 indicating high fear of movement. The TSK-Dutch version that will be used in this study is shown reliable and valid [74,80-82].

The Illness Perception Questionnaire-Revised (IPQ-R), consisting of 3 domains, will be used to measure patients’ illness perceptions [83]. In the first domain, called illness identity, the perceived symptoms and their possible relation to the illness are evaluated. In this study, participants will indicate whether or not they believe that a specific symptom is related to spinal pain (“yes” or “no”). The second domain, the beliefs domain, covers 7 dimensions: the acute/chronic timeline, the cyclical character of the illness, the consequences, controllability, curability, emotional representations and illness coherence. The third domain lists 18 possible causes to which individuals attribute their condition, the degree to which individuals perceive themselves as responsible for the illness, as well as the responsibility individuals take for curing themselves. For each item in the second and third domain, patients rate their level of agreement on a 5-point Likert scale, ranging from “strongly disagree” to “strongly agree” [83]. Studies have shown that both the English and Dutch versions of the IPQ-R have excellent validity and reliability [84,85].

### Interventions

Before starting the study, physiotherapists involved in the treatment will be trained extensively by expert therapists in the domain. They will also receive a manual containing descriptions of procedures and checklists. Using a therapists’ treatment diary, therapy will be monitored and evaluated.

Within a 12-week period, patients in each group will receive 18 treatment sessions from their trained physiotherapist. In both groups, the first 3 sessions (spread over 2 weeks) will consist of education, whereas the next 15 sessions (spread over the next 10 weeks) will be exercise according to the protocol. All sessions are one-on-one sessions lasting about 30 minutes, except for session 1 (group session, maximum 6 persons/group, 1 hour) and session 2 (online module performed at home).

The educational information will be presented verbally (explanation by the therapist) and visually (summaries, pictures, and diagrams on computer). After the first session, patients will also receive an information leaflet about the education according to the protocol and will be asked to read it carefully. Although initiated during the first 3 sessions, education will be ongoing throughout exercise therapy. In addition to the individually tailored exercises performed in physiotherapy, a home exercise program will be established for each patient. For home exercises, modalities and clear verbal, written and visual instructions will be given. Patients will be strongly encouraged to continue these exercises during the follow-up year.

### Modern neuroscience group

The modern neuroscience approach will entail therapeutic pain neuroscience education followed by cognition-targeted motor control training. Pain neuroscience education will be applied to reconceptualize pain and to convince the patients that all pain is in the brain, and that hypersensitivity of the central nervous system rather than local tissue damage may be the cause of their symptoms. The education will cover the physiology of the nervous system in general and of the pain system in particular. The content and pictures of the educational sessions are based on the book “Explain Pain” [86] and have been used in earlier pilot studies [29,30,87,88].

In the present study, the Dutch Neurophysiology of Pain Test (patient version) [89] will be used as part of the intervention to ascertain the quality of the education program: after the third session, patients will be asked to fill out this valid and reliable questionnaire to assess their knowledge on pain neurophysiology [89,90]. 90% of the patients should pass the test (desired mean score of 65%). Patients’ misinterpretations will be discussed further upon completion of the questionnaire.

As such, therapeutic pain neuroscience education (or rather “communication”) will prepare the patients for a time-contingent, cognition-targeted approach to daily (physical) activity and exercise therapy. Once adaptive beliefs are acquired regarding CSP, the exercise therapy with specific emphasis on spinal motor control training will be initiated (session 4). This training will consist of sensorimotor control training by facilitating the proprioceptive system and optimizing the coordinative muscle recruitment patterns [68,91-93]. However, some modifications will be made to the original motor control program to comply with modern pain neuroscience (i.e., cognition-targeted motor control training detailed in [22,30]). In neck pain patients, this phase of the exercise will involve retraining of the deep cervical flexors/extensors and scapular muscles, whereas retraining of the deep muscles surrounding the lumbopelvic region (e.g., multifidus, transversus abdominis, psoas, pelvic floor muscles) will be performed in patients with low back pain. Progression to a next level of (more difficult) dynamic and functional exercises will be preceded by an intermediate phase of motor imagery [22,30].

A time-contingent progression will be used to integrate exercises with increasing complexity. In order to maximize transfer to daily situations, late-stage progression will not only involve exercising during physically demanding tasks, but also exposure to the feared movements or activities, and exercising during cognitively and psychosocially stressful conditions [22,30]. Throughout the cognition-targeted motor control training program, patients’ cognitions and perceptions about their problem and about exercises will be addressed.

Further details of the modern neuroscience approach to CSP, including practice guidelines, were presented previously [22,94,95].

### Control group

Those in the control group will receive traditional back/neck school, including back care education and general exercises. Back care education will cover anatomy and biomechanics of the spine, common causes of spinal pain, the load-tolerance model, nociceptive pain processing, and ergonomic counseling based on the inherent postural strain associated with various postures and daily activities (including standing, sitting, and lifting). As such, the education sessions will prepare the patients for a symptom-contingent, biomedical approach to daily (physical) activity and exercise therapy. In session 4, the general exercise therapy will be started with specific emphasis on treating dysfunctional muscles and joints. Different therapeutic goals will be pursued (e.g. microcirculation, mobility, endurance, strength) depending on what emerges from the clinical reasoning as the most dominant peripheral dysfunction. Importantly, abdominal and paraspinal muscles will be targeted without involvement of deep muscle activation. The program will also involve aerobic fitness improving exercises. The progressive exercise program will mainly entail an increase in exercise intensity, and an evolution towards functional activities and more physically demanding tasks while keeping the spine in physiological neutral positions to minimize strain imposed upon the spinal structures. All exercises will be performed in a symptom-contingent way.

### Main treatment contrast

The main difference between the 2 groups is the treatment of cognitive aspects of pain in the modern neuroscience group (biopsychosocial approach), which will not be applied in the control group (biomedical approach, symptom-contingent treatment).

### Sample size calculation

Sample size calculations were performed with G*Power 3.1.3 (Düsseldorf, Germany) based on the therapy effects on pain in the pilot study of Moseley [29], and accounting for a 30% loss to follow-up after 1 year. Calculations were based on one-tailed testing with alpha set at 0.01 and a desired power of 0.95. Allocation ratio (N2/N1) was defined as 1, resulting in 60 patients in the experimental group and 60 in the control group.

### Data analysis

Data analysis will be performed using SPSS for Windows (version 22, SPSS Inc; Chicago, IL), or the newest available version, under the intention-to-treat-principle. Baseline data will be analyzed in order to determine descriptive statistics for the different outcome measures for the complete CSP group. Comparability of the groups before the intervention will be studied with the Fisher exact test and independent samples *t* test. Associations between baseline parameters will be examined. Possible changes in the outcome measures in response to the intervention will be examined between the two groups by using repeated measures analysis of variance with intervention serving as the between-subjects factor and time as the within-subjects factor. In these analyses, treatment center, dominant pain location and gender will be entered as covariates. Regression analyses will be used to determine predictors for therapy success and reasons for poor therapy outcome. For all statistical tests, the significance level will be set at 0.05. Risk ratios and their 95% confidence intervals will be calculated as are effect sizes [96]. The number needed to treat and its 95% confidence interval will be calculated for the outcomes in which a beneficial effect of the experimental treatment is achieved. In case of adverse effects, the number needed to harm and its 95% confidence will also be calculated.

### Blinding

The present study is a triple-blind randomized controlled trial. The patient, assessor and outcomes assessor will be blind to the treatment groups. To keep patients unaware of any expected treatment group benefit, patients will be informed that the effect of 2 well-established therapies is to be evaluated. An independent and blinded assessor will perform the baseline and follow-up assessments. Statistical analysis will be blinded regarding treatment group code. The researcher who will perform the statistical analyses will not be involved in taking the measurements. The treating physiotherapists will be blinded to the results of the measurements and questionnaires.

### Ethics

This trial will be conducted in compliance with the Declaration of Helsinki (1964 and amendments) and Good Clinical Practices. Patients will give their written informed consent prior to the start of any study-related procedure. Approval to conduct this study was granted by the Ethics Committee of the Ghent University Hospital and the University Hospital Brussels.

### Results

Inclusion of patients began in February 2014 and is expected to last until March 2016. Results are expected in 2017.

## Discussion

The aim of this study is to compare usual care physiotherapy and a modern neuroscience approach in CSP patients. The main study question is which of the 2 treatment strategies is more effective in reducing pain and disability associated with CSP, both in the short and long term (1-year follow-up). Further objectives are: to evaluate the effect on brain gray matter structure, the factors associated with therapy success, the relationships between (changes in) outcome parameters being pain, disability, brain structure, motor control, muscle properties and psychosocial correlates.

With the inclusion of 120 patients with CSP, this will be the largest study to investigate the effectiveness of 2 well-founded physiotherapy treatment strategies for patients with CSP. Furthermore, the multi-center design of the study increases the external validity of the study findings as it implies treatment by different physiotherapists in different settings and a large geographical area for patient recruitment. Importantly, the treating physiotherapists, blinded to the results of the measurements and questionnaires, are equally instructed and experienced in applying the respective treatments. Randomization is organized centrally by the Biostatistics Unit and the randomization schedule is only known to 1 investigator who is not involved in recruiting patients. The patients, assessors performing the baseline and follow-up evaluations and the researcher performing statistical analyses are blinded to group allocation. Hence, the study is designed in a way that minimizes potential biases.

It is expected that this randomized controlled trial will provide novel data on the effectiveness of a modern neuroscience approach when compared to usual care physiotherapy on key patient-centered outcome measures (i.e., pain and disability). These results will also contribute to understanding the associations between (changes in) pain, disability, psychosocial correlates and physical factors, including brain structure. Moreover, the study will provide insights into major factors associated with (sub) optimal treatment success. As such, this 12-month prospective trial may contribute towards refining guidelines for good clinical practice and may be used as a basis for health authorities’ recommendations.

## Abbreviations

COP: Centre of pressure; CSP: Chronic spinal pain; CPT: Cold pressor test; CSI: Central Sensitization Inventory; IPQ-R: Illness Perception Questionnaire-Revised; MR: Magnetic resonance; MRI: Magnetic resonance imaging; NRS: Numerical Rating Scale; PCS: Pain Catastrophizing Scale; PDI: Pain Disability Index; PPT: Pressure pain threshold; PVAQ: Pain Vigilance and Awareness Questionnaire; SF-36: Medical outcomes Short Form 36 Health Service Survey; TSK: Tampa Scale for Kinesiophobia.

## Competing interests

The authors declare that they have no competing interests.

## Authors’ contributions

All authors contributed to the design of the study and have read and approved the final manuscript.

## Pre-publication history

The pre-publication history for this paper can be accessed here:

http://www.biomedcentral.com/1471-2474/15/149/prepub
